# Graph Eigen Decomposition-Based Feature-Selection Method for Epileptic Seizure Detection Using Electroencephalography †

**DOI:** 10.3390/s20164639

**Published:** 2020-08-18

**Authors:** Md. Khademul Islam Molla, Kazi Mahmudul Hassan, Md. Rabiul Islam, Toshihisa Tanaka

**Affiliations:** 1 Department of Computer Science and Engineering, University of Rajshahi, Rajshahi 6205, Bangladesh; 2 Department of Computer Science and Engineering, Jatiya Kabi Kazi Nazrul Islam University, Trishal, Mymensingh 2224, Bangladesh; kmhassan@jkkniu.edu.bd; 3 Institute of Global Innovation Research, Tokyo University of Agriculture and Technology, Tokyo 184-8588, Japan; rabiul@sip.tuat.ac.jp; 4 Department of Electrical and Electronic Engineering, Tokyo University of Agriculture and Technology, Tokyo 184-8588, Japan; 5 RIKEN Center for Advanced Intelligence Project, Tokyo 103-0027, Japan

**Keywords:** discrete wavelet transform, electroencephalography, feature selection, epilepsy, seizure

## Abstract

Epileptic seizure is a sudden alteration of behavior owing to a temporary change in the electrical functioning of the brain. There is an urgent demand for an automatic epilepsy detection system using electroencephalography (EEG) for clinical application. In this paper, the EEG signal is divided into short time frames. Discrete wavelet transform is used to decompose each frame into a number of subbands. Different entropies as well as a group of features with which to characterize the spike events are extracted from each subband signal of an EEG frame. The features extracted from individual subbands are concatenated, yielding a high-dimensional feature vector. A discriminative subset of features is selected from the feature vector using a graph eigen decomposition (GED)-based approach. Thus, the reduced number of features obtained is effective for differentiating the underlying characteristics of EEG signals that indicate seizure events and those that indicate nonseizure events. The GED method ranks the features according to their contribution to correct classification. The selected features are used to classify seizure and nonseizure EEG signals using a feedforward neural network (FfNN). The performance of the proposed method is evaluated by conducting various experiments with a standard dataset obtained from the University of Bonn. The experimental results show that the proposed seizure-detection scheme achieves a classification accuracy of 99.55%, which is higher than that of state-of-the-art methods. The efficiency of FfNN is compared with linear discriminant analysis and support vector machine classifiers, which have classification accuracies of 98.72% and 99.39%, respectively. Hence, the proposed method is confirmed as a potential marker for EEG-based seizure detection.

## 1. Introduction

Seizure is a central nervous system disorder in which brain activity becomes abnormal. The changes in the brain’s electrical activity can cause dramatic, noticeable symptoms or, in other cases, no symptoms at all. Sometimes, seizure becomes the source of unusual behavior, sensations, and loss of awareness [1]. The number of seizure patients globally indicates the need for a robust automatic seizure-detection technique. Recently, automatic detection of seizure events using efficient algorithms has become a challenging task for the biomedical engineering community. Electroencephalography (EEG) is an effective diagnostic modality for epilepsy seizure detection. In practice, to detect seizures based on EEG signals, epileptologists need to observe long-term multichannel EEG signals, which is tedious and cumbersome. Therefore, an efficient algorithm for detecting seizures based on long-term multichannel EEG signals would be invaluable for epileptologists. EEG records electrical activities of the brain containing potentially pathological information. An epileptiform pattern that is effectively used for epileptic-seizure detection is the presence of spikes in EEG signals [2,3]. This pattern has become a valuable tool for assessing brain disorders, especially epileptic seizure [4]. An automatic system for seizure detection makes a significant contribution to long-term epilepsy monitoring, as well as to rehabilitation and diagnosis [5]. Various feature parameters have been proposed for the analysis of EEG signals. Notably, most existing methods rely on decomposing the EEG signal into several levels to attain better classification results [6]. In this study, the EEG signal is decomposed into a set of narrowband signals from which a number of features are extracted. The dominant features are then selected from the training data. The seizure event is detected in the EEG using an artificial neural network.

The rest of the paper is organized as follows. Previous research related to the study is discussed in Section 2. Section 3 describes the dataset used in this study. The proposed methodology is presented in Section 4, and the experimental results are presented in Section 5. Section 6 offers a discussion of the proposed work and its comparison with state-of-the-art methods. Section 7 concludes with overall remarks.

## 2. Related Works

The detection of seizure events in EEG signals is a classification problem. It involves the extraction of the discriminative features from an EEG signal in order to perform classification. Several researchers have proposed feature-extraction methods for the automated detection of epileptic seizure. It is necessary to review the existing methods to understand the importance of the current state-of-the-art, future trends, and limitations of existing approaches. The following paragraphs provide an overview of the related state-of-the-art methods for the classification of seizure and nonseizure EEG signals.

The EEG signal is nonlinear and nonstationary [7]. A two-stage hybrid system with feature extraction using fast Fourier transform (FFT) and decision-making using a decision tree classifier has been implemented for seizure detection, considering that EEG is stationary in short duration [8]. Although the method has achieved 98.72% classification accuracy, it is not compliant with EEG characteristics. The Fourier-transform-based time–frequency (t–f) representation has been performed, considering that EEG is nonstationary in short duration [9]. The fractional energy of each t–f window is used as a feature, and the classification has been accomplished using neural network, which has achieved 89.1% average classification accuracy. The wavelet transform [10] and multiwavelet transform [11] are also potential approaches to signal decomposition, leading to the extraction of features from each subband of the EEG for seizure detection. A variety of methods have been developed for epilepsy recognition using entropy-based features derived from the EEG signal [11,12,13,14,15].

In [11], multiple orthogonal and symmetric wavelet functions have been used to decompose the EEG signal into multiple subbands. The approximate entropy (APE) has been extracted from each subband. An artificial neural network has been used for seizure detection using the subband APE with a classification accuracy of 98.27%. The entropy computed from wavelet packet coefficients has been effectively used in epilepsy recognition, with an average classification accuracy of 99.44% [12]. Automated epileptic seizure detection has been effectively implemented with a support vector machine (SVM) using the permutation entropy (PE) of a short-term EEG segment, achieving 86.10% accuracy [13]. Notably, PE has a lower value for epileptic EEG than for nonepileptic EEG. The SVM has also been utilized for seizure event recognition using wavelet-decomposition-based subband fuzzy APE (fAPE) [14] and weighted PE (WPE) [15] as potential features. The abrupt change in the EEG signal is tracked effectively by WPE. The value of fAPE for epileptic segment of EEG is higher than that of the normal EEG segment. The classification accuracies of subband fAPE and WPE are 98.45% and 93.37%, respectively. The entropy-based feature is not effective in all respects for characterizing spikes in EEG for epilepsy detection, and hence it is necessary to introduce other features.

The Burg autoregressive (AR) coefficients have been used as features for epilepsy detection in [16], considering that the short-term frame of EEG is stationary. The least square SVM (LS-SVMs) has been implemented in epilepsy classification with an accuracy of 99.56%. A seizure-detection method has been implemented in [17] with genetic programming (GP)-based feature extraction and a *k*-nearest neighbors (KNN) classifier. An ensemble model of pyramidal one-dimensional convolutional neural network (P-1D-CNN) has been used in [18] for seizure detection. Learning with a lower number of parameters has been implemented with this model, attaining 99.10% classification accuracy. Learning-based feature extraction requires a high volume of training data. In [6], the matrix determinant of EEG has been used as a significant feature for epilepsy recognition. The artifact-free filtered EEG time series has been arranged sequentially to form a square matrix with which to compute the matrix determinant feature, and a multilayer perceptron has been employed as the classifier, with an accuracy of 97.15%. A Gabor filter bank has been used to derive 1D-LBP (local binary pattern)-based features in [19], and a KNN classifier is utilized to recognize seizure events in recorded EEG. The improved correlation-based feature selection (ICFS) method has been proposed by Mursalin et al. [20] to detect epileptic seizure with a random forest (RF) classifier. The entropy-based features, as well as different time and frequency domain features, have been used. The prominent feature selection has been performed by applying ICFS. A set of 28 features have been extracted from time, frequency, and statistical domains, and significant features have been selected using neighborhood component analysis (NCA) [21]. In NCA, optimization of regularization parameters has ensured better classification accuracy (less classification loss) with seven features, with an accuracy of 96.1% using the Bern–Barcelona dataset. The feature-selection approach requires a high volume of labeled data for the purpose of training.

Empirical mode decomposition (EMD) has been used to analyze nonstationary and nonlinear signals. It has also been used in the classification of epileptic seizures recorded by EEG [22]. EMD decomposes any signal into a finite set of basis functions called intrinsic mode functions (IMFs). The ellipse area of the second-order difference plot (SODP) of selected IMF(s) has been used as the seizure-detection feature [7], with an accuracy of 97.75%. The signal-processing task using EMD requires a computational cost higher than Fourier and wavelet-based approaches. Moreover, EMD is a nonconsistent form of decomposition. It may generate a different number of IMFs while decomposing the same signal at different times. A set of selected IMFs has been used to detect seizure in [7]. The selection of the appropriate IMFs to obtain maximum classification accuracy has been performed heuristically, and hence it is difficult to reproduce the results.

In this study, discrete wavelet transform (DWT) is used to decompose the analyzing EEG signal into a set of subbands. A set of significant features extracted from all the subband signals is utilized in the seizure classification scheme. All the subbands obtained by DWT are used to extract features. In addition to the subbands, the same features are extracted from the EEG signal. Here, three features are used to characterize the spike events in the EEG signal. It is well known that an EEG signal indicating a seizure event has more spike events than a nonseizure EEG signal [2]. Moreover, several entropy-related features are utilized to quantify the complexity of EEG signals for characterizing the seizure event. It has been reported that the combination of different entropies of EEG signal improves seizure-detection performance [23,24,25]. Therefore, four entropy-based features are employed here. The seven features are extracted from each subband and concatenated to obtain the feature vector. The combination of spike-detecting features, as well as entropy features, has a significant ability to effectively distinguish an EEG signal with a seizure event from a normal one. Feature selection is essential to improving the performance of the machine-learning-based classification approach [26]. Its objective is the selection of a minimal feature subset that permits a problem to be defined clearly. By selecting a minimal subset of features, the redundant and irrelevant features are removed based on the criterion that the original task is achieved equally well, if not better. It reduces the computational cost as well. An effective feature-selection approach potentially plays the role of improving the classification performance [27,28,29]. Here, a graph-based eigen decomposition approach is implemented to select the potential features from a high-dimensional feature vector for seizure detection. The selected features are used to classify EEG signals into seizure and nonseizure categories using a feedforward neural network (FfNN). The narrowband features of the EEG signal are used as effective features for seizure detection.

## 3. Data Description

The EEG dataset provided by the Department of Epileptology, University of Bonn, Germany, is used to evaluate the performance of the proposed method [30]. It is publicly available and consists of five sets of EEG data, labeled A, B, C, D, and E. Each of the five sets contains 100 segments of single-channel EEG of 23.6 s duration. The segments were collected from continuous recordings of multichannel EEG. Visual inspection was performed in prior for artifacts of eye movement and other muscle activities. Surface EEG recordings were carried out for sets A and B on five healthy volunteers using a standardized electrode placement scheme during their awake state with eyes open (A) and eyes closed (B), respectively. Sets C, D, and E were collected from EEGs of presurgical diagnosis. The EEGs from five patients were selected. All had achieved complete seizure control after resection of one of the hippocampal formations, which was thus correctly diagnosed as the epileptogenic zone. The EEGs in sets C and D were recorded from the hippocampal formation of the opposite hemisphere and within the epileptogenic zone of the brain. In turn, sets C and D contained the activity measured during seizure-free intervals, and set E contained only seizure activity. A summary of the EEG dataset is shown in Table 1.

**Figure 1:**
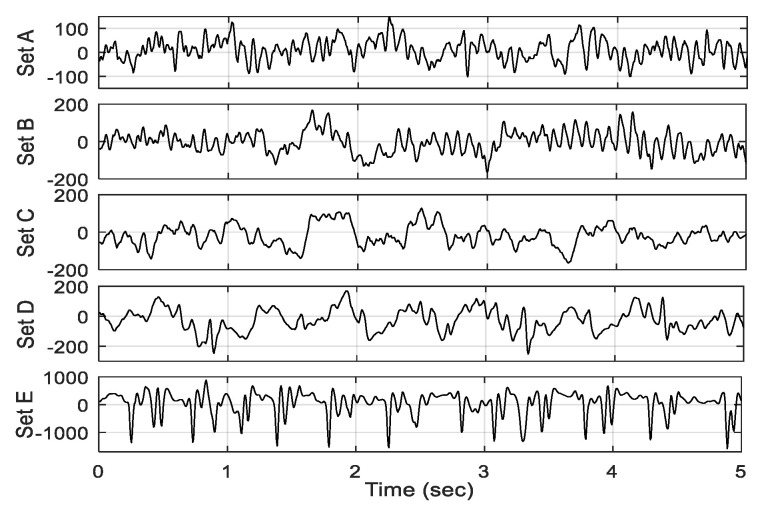
Electroencephalography (EEG) subframes selected from each of five sets (A–E).

All EEG signals were recorded using the same 128-channel amplifier system with an average common reference. The data were discretized at a sampling rate of 173.61 Hz and 12-bit analog-to-digital conversion. A band-pass filter with frequency range 0.53–40 Hz was used to preprocess the data. As examples, EEG frames of 5 s length selected from each of the five sets (sets A–E) are illustrated in Figure 1.

## 4. Methodology

**Figure 2:**
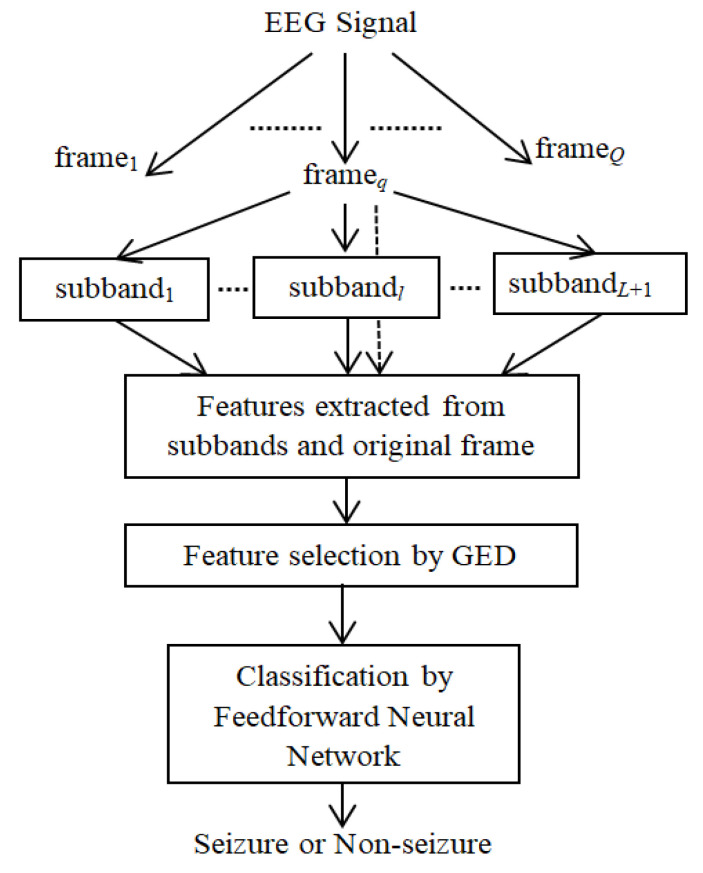
Block diagram of the proposed method (GED: graph eigen decomposition).

Here, a novel method is proposed for subband feature extraction: potential feature selection to classify epileptic and nonepileptic EEG signals using a neural network. The block diagram of the method is shown in Figure 2. It has the following steps:

(i) The segment of the EEG signal is divided into fixed length frames.
(ii) Each frame is decomposed into a number of subband signals using DWT.
(iii) Three spike-related features, namely, ellipse area of SODP, fluctuation index, and squared coefficient of variation of the absolute series, as well as four entropy features, are computed from each subband of a frame. The same features are also extracted from the original frame before subband decomposition.
(iv) All the extracted features of a frame are combined to derive the feature vector.
(v) A graph eigen decomposition (GED)-based approach is implemented to select the discriminative features.
(vi) An FfNN is trained with the selected features, and classification of the EEG frame into ictal and interictal categories is performed.

Each of the five sets of EEG data has 100 segments of 23.6 s length. The framing of each EEG segment is performed with an overlapping of reasonable portion. On the basis of the requirement of the medical application, the size of the frame is set at 10 s with 50% overlapping. The EEG frame of 10 s length is decomposed into subband signals using DWT. The desired features are extracted from each subband as well as each EEG frame. All the features (obtained from subbands and EEG frames) are combined to derive the raw feature vector corresponding to each frame. The proper selection of dominant features enhances classification accuracy in the machine learning paradigm. A GED-based method is implemented to select the potential feature subset. It suppresses the redundant features that have no significant role in epilepsy classification. Thus, the significant features of the training dataset obtained are used to train the FfNN classifier. Then, the performance is evaluated by the test dataset. The following subsections describe the steps of the proposed seizure-detection method.

### 4.1. Subband Decomposition

**Figure 3:**
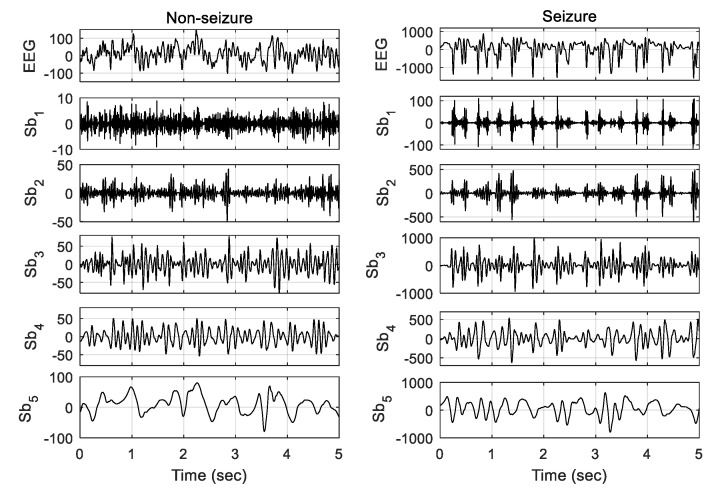
The EEG signals and their different subbands obtained by applying four levels of DWT. Left column: nonseizure EEG signal (from set A) and its five subbands; right column: seizure EEG signal (from set E) and its five subbands. Note that the signals of 5 s length (out of 10 s frames) are plotted here for better illustration.

The narrowband features provide discriminative information for seizure detection [12,20]. EEG is characterized as a nonstationary signal, and hence Fourier transformation is not an appropriate tool for analyzing it. Most physiological signals with a nonstationary pattern are effectively analyzed by DWT [31]. It is effectively used for subband decomposition of EEG frames. The method is based on a filter bank decomposition on an orthogonal base realized by a convolution between the original signal *x*(*n*) and the filter. There are two filters: the highpass filter (*g*), which computes the detail coefficient *d^l^*(*n*); and the lowpass filter (*h*), which computes the approximate coefficient *a^l^*(*n*) at decomposition level *l*. The convolution realized by the highpass and lowpass filters is $d^{l}\left(n\right)=\underset{k}{\sum }g\left(k\right)*a^{l-1}\left(2n-k\right)$ and $a^{l}\left(n\right)=\underset{k}{\sum }h\left(k\right)*a^{l-1}\left(2n-k\right)$, respectively, where *l* is the current wavelet decomposition level, *n* is the number of time observations, and *g*(*k*) and *h*(*k*) are the filter coefficients of the lowpass and highpass filters. Note that both approximation and detail coefficients at level *l* depend only on the approximation coefficient at level (*l* − 1). The db4 wavelet function is utilized for EEG decomposition into subbands. The one approximate and *L* detail coefficients are obtained by DWT with *L* level of decomposition [28]. The subband signals are reconstructed using the coefficients. Hence, (L+1) subbands’ (*s*_1_, *s*_2_,…, *s*_*L*+1_) signals are obtained by *L* levels of wavelet decomposition. The epileptic and nonepileptic EEGs and their five subbands obtained by four levels of wavelet decomposition are shown in Figure 3. The cutoff frequencies of the five subbands Sb1, Sb2, Sb3, Sb4, and Sb5 are 43–86 Hz, 22–43 Hz, 11–22 Hz, 6–11 Hz, and 0–6 Hz, respectively. The first subband Sb1 (43–86 Hz) corresponds to the gamma rhythm. None of other four subbands relate to a single rhythmic component, but a combination of the parts of two rhythms. The second subband Sb2 (22–43 Hz) includes the high frequency part of beta rhythm in addition to gamma, Sb3 (11–22 Hz) contains the low frequency part of beta as well as high frequency part of alpha rhythm, Sb4 mostly includes the mu band, and Sb5 comprises the low frequency part of theta in addition to delta rhythm. The high-frequency ripples contain significant information for seizure detection. Although the gamma rhythm (>30 Hz) has a substantial role in seizure detection, the other rhythms also carry important evidence related to the same task. The four-level DWT decomposition is used in this study. The experimental validation of the number of decomposition level is illustrated in Section 5.

### 4.2. Feature Extraction

A feature is an individual measurable property or characteristic of an object. The extraction of informative, discriminating, and independent features is a crucial step for effective algorithms of pattern classification in machine learning. The feature-extraction method is useful for reducing the volume of data required for processing without losing important or relevant information. It reduces the amount of redundant data for a given analysis. Feature extraction is a general term for methods of constructing combinations of variables to get around these problems while still describing the data with sufficient accuracy. The features are very much application-dependent. Two broad types of features are used in this study. The first three features illustrate the spike characteristics of the EEG signals, and the other group of four entropy-based features represents the complexity of the signal. The combination of these two categories of features is used to characterize the EEG signals with respect to seizure events. The features are described immediately below.

#### 4.2.1. Ellipse Area of SODP

The second-order difference plot (SODP) is a graphical representation of successive increments against each other, and it provides the signal’s rate of variability. It is widely used to determine the spike events of a signal. The SODP of signal *x*(*n*) is a graph of *y*_2_(*n*) against *y*_1_(*n*), which are defined as [7]

*y*_1_ = *x*(*n* + 1) − *x*(*n*) (1)

*y*_2_ = *x*(*n* + 2) − *x*(*n* + 1) (2)

The lengths of *x*(*n*), *y*_1_(*n*), and *y*_2_(*n*) are *N*, (*N* − 1), and (*N* − 1), respectively. 

**Figure 4:**
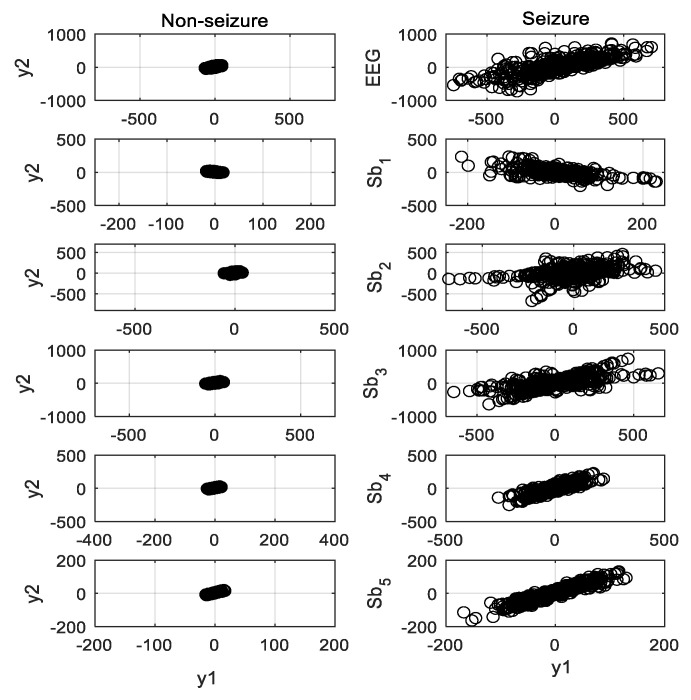
Second-order difference plot (SODP) of five subbands obtained from nonseizure (**left**) and seizure (**right**) EEG signals.

Figure 4 shows the SODP of seizure and nonseizure EEG signals and their five subbands illustrated in Figure 3. The area of the ellipse covering the SODP is calculated for the plots. The epileptic EEG has a higher number of transient events, and hence provides a bigger elliptical structure than the seizure-free signal. The 95% confidence area is used to determine the ellipse area occupied by the data points of the subband signal [24,32]. The ellipse area *A_e_* of SODP with 95% confidence is defined as [7]

*A_e_* = π*αβ*, (3)

where *α* and *β* are the major and minor radii of the 95% confidence ellipse area, respectively, and the terms are defined as follows:

(4) $$\alpha =\sqrt{3(\kappa_{1}^{2}+\kappa_{2}^{2}+\delta )}$$

(5) $$\beta =\sqrt{3(\kappa_{1}^{2}+\kappa_{2}^{2}-\delta )}$$

(6) $$\delta =\sqrt[{}]{\left(\kappa_{1}^{2}+\kappa_{2}^{2})-4(\kappa_{1}^{2}\kappa_{2}^{2}-\kappa_{12}^{2}\right)}$$

Here, δ represents dispersion for the radii *α* and *β*. The parameters $\kappa_{1}$ and $\kappa_{2}$ represent the root mean squares of *y*_1_ and *y*_2_, respectively. The term $\kappa_{12}$ is the covariance of *y*_1_ and *y*_2_. These parameters are calculated as

(7) $$\kappa_{1}=\sqrt[{}]{\frac{1}{N-1}\sum_{n=1}^{N-1}y_{1}{\left(n\right)}^{2}}$$

(8) $$\kappa_{2}=\sqrt[{}]{\frac{1}{N-1}\sum_{n=1}^{N-1}y_{2}{\left(n\right)}^{2}}$$

(9) $$\kappa_{12}=\;\frac{1}{N-1}\;\sum^{\;}y_{1}\left(n\right)y_{2}\left(n\right)$$

As already illustrated in Figure 4, the spread area of the second order plot for the ictal EEG is higher than that for the interictal signal. The epileptic EEG signal produces a larger elliptical structure than the nonepileptic signal. In addition to the subbands’ (narrowband) signals, the ellipse area of SODP of fullband EEG also carries significant discriminative information for seizure detection. It motivates the use of the SODP ellipse area of the EEG signal, including its subbands, as a potential feature for seizure detection.

#### 4.2.2. Squared Coefficient of Variation of the Absolute Series 

The squared coefficient of variation of the absolute series denoted by *V_sc_* provides a quantitative measure of the amount of amplitude fluctuation in any signal *x*(*n*). The seizure EEG signal has a higher number of spikes and fluctuations in amplitude, which results in a larger value of *V_sc_* compared with the nonepileptic signal. It is defined as [4]

(10) $$V_{sc}=\frac{\sigma^{2}}{\mu^{2}}$$

where $\mu =\frac{1}{N}\overset{N}{\underset{n=1}{\sum }}\left|x\left(n\right)\right|$ and $\sigma =\sqrt{\frac{1}{N}\overset{N}{\underset{n=1}{\sum }}{\left(x\left(n\right)-\frac{1}{N}\overset{N}{\underset{n=1}{\sum }}x\left(n\right)\right)}^{2}}$ for any signal *x*(*n*) of length *N*. The value of the squared coefficient of variation of the absolute series for the epileptic EEG signal is supposed to be larger than that for the nonepileptic EEG signal.

#### 4.2.3. Fluctuation Index

The fluctuation index (*F_i_*) measures the degree of the changes in the amplitude of any signal. The value of *F_i_* refers to the amount of frequent changes in the amplitude of a signal. The epileptic EEG has frequent changes in amplitude, yielding a higher value of *F_i_* compared with the nonepileptic EEG. It is measured by the mean absolute of all first-order increments of *x*(*n*) and defined as [4]

(11) $$F_{i}=\frac{1}{N-1}\sum_{n=1}^{N-1}\left|x\left(n+1\right)-x\left(n\right)\right|,$$

where *x*(*n*) is the analyzed signal of length *N*.

#### 4.2.4. Permutation Entropy

The complexity of a time series is estimated by permutation entropy (PE). It is a simple and robust method used for automated seizure detection [33]. For any time series *x*(*n*), each vector *X*(*k*) can be represented as X(k)=[x(k), x(k+τ), …, x(k+(δ−1)τ)], for *k* = 1, 2, …, *N* − *δ* + 1, where *δ* and *τ* represent the embedding dimension and time lag, respectively. The permutation of [1, 2, …, *δ*] can be defined as $П=\left[j_{1},\;j_{2},\;\ldots ,\;j_{\delta }\right]$, which satisfies $x\left(n+\left(j_{1}-1\right)\tau \right)\leq \;x\left(n+\left(j_{2}-1\right)\tau \right)\;\leq \;\ldots \;\leq \;x\left(n+\left(j_{\delta }-1\right)\tau \right)$. The probability of each possible permutation ${П}_{l}(l=\;1,2,\ldots ,\;\delta !$) for the set of vectors ${\left\{X\left(k\right)\right\}}_{k=1}^{N-\left(\delta -1\right)\tau }$ can be estimated as $p\left({П}_{l}\right)=\;C\left({П}_{l}\right)/\left(N-\left(\delta -1\right)\tau \right)$, where *N* is the length of the analyzing signal *x*(*n*) and $C\left({П}_{l}\right)$ represents the number of occurrences of the order pattern ${П}_{l}$. The PE can be defined as

(12) $$pE=\;-\sum_{l=1}^{\delta !}p\left({П}_{l}\right)\log p\left({П}_{l}\right),$$

In this study, the parameters *δ* and *τ* were set to 3 and 1, respectively [34,35]. Several studies in epilepsy seizure detection were suggested to use the value of the parameter *δ* from 3 to 7 [13,33,36,37]. Sharma et al. proposed an automatic epileptic seizure detection system with entropy measures [36]. Their used parameters were *δ* = 3 and *τ* = 1 by motivating the previous epilepsy studies [13,33,37].

#### 4.2.5. Approximate Entropy

Approximate entropy (APE) was first proposed by Pincus et al. [38] to measure the amount of regularity in a time series. APE is extensively used in many areas of biomedical signal processing, including EEG and ECG signals. For any time series *x*(*n*) with length *N*, we can define the set of vectors as ${\left\{X\left(k\right)\right\}}_{k=1}^{N-\delta +1}$, and each *X*(*k*) vector can be represented as

(13) X(k)=[x(k), x(k+1),…,x(k+δ−1)] for 1≤k ≤N−δ+1,

where *δ* is the embedding dimension. In recent epilepsy studies [34,39], the value of *δ* for approximate entropy was chosen as 2 for designing the automatic system. In this work, we have taken *δ* = 2 for the computation of APE. To measure the complexity of a time series, the approximate entropy is defined as

(14) $$apE\left(\delta ,\;\alpha ,\;N\right)=\;\frac{1}{N-\delta +1}\;\sum_{k=1}^{N-\delta +1}\log\left(C_{k}^{\delta }\left(\alpha \right)\right)-\;\frac{1}{N-\delta }\;\sum_{k=1}^{N-\delta }\log\left(C_{k}^{\delta +1}\left(\alpha \right)\right),$$

where *α* defines the tolerance limit with 0.2 times the standard deviation of data used in different studies [24], and $C_{k}^{\delta }\left(\alpha \right)$ is the correlation integral and is defined as

(15) $$C_{k}^{\delta }\left(\alpha \right)=\;\frac{1}{\left(N-\delta +1\right)}\;\sum_{j=1}^{N-\delta +1}I\left(D\left(X\left(k\right)-X\left(j\right)\right)\leq \;\alpha \right),$$

where I(·) represents the indicator function and the *D*(·) is the distance between two vectors *X*(*k*) and *X*(*j*).

#### 4.2.6. Renyi’s Entropy

The Renyi’s entropy has been used for seizure classification purposes in [40]. It generalizes the Shannon entropy and offers a more flexible tool, allowing for a better characterization of the process than just the Shannon entropy [41]. Several studies related to epileptic seizure detection have proposed the use of Renyi’s entropy [40,42,43,44]. Let *X*(*f*) for *f* = 1, 2, …., *F* be the Fourier transform of the signal *x*(*n*) of length *N*. The normalized power spectral density *p_f_* can be estimated as

(16) $$p_{f}=\frac{{\left|X\left(f\right)\right|}^{2}}{\sum_{f=1}^{F}{\left|X\left(f\right)\right|}^{2}}$$

where *F* is the number of frequency bins (here, *F* = *N*/2). The Renyi’s entropy with order-α [34,35] can be calculated as

(17) $$enE\left(\alpha \right)=\;\frac{1}{1-\alpha }\log\sum_{f}{\left(p_{f}\right)}^{\alpha }$$

Here, we use only *α* = 2 [24,36,40,44], while omitting a discussion of other orders of the Renyi’s entropy.

#### 4.2.7. Phase Entropy

Phase entropy is defined based on the bispectrum, the simplest case of higher-order spectra [23]. The bispectrum of a time series can be defined as

(18) $$\partial \left(f_{1},f_{2}\right)=E\left[X\left(f_{1}\right)X\left(f_{2}\right)X^{*}\left(f_{1}+f_{2}\right)\right],$$

where X(f) (*f* = 1, 2, …, *F*) represents the Fourier transform of signal x(n) of length *N*, *F* is the number of frequency bins (here, *F* = *N*/2), and E represents the expected value. The terms $f_{1}$ and $f_{2}$ represent the $f_{1}{}^{th}$ and $f_{2}{}^{th}$ frequency bins of X(f), respectively, and ($f_{1}+f_{2})$ is the harmonic component of $f_{1}$ and $f_{2}$. Then, the phase entropy *phE* of x(n) can be defined as

(19) $$phE=\;-\sum_{f_{1}=1}^{F}\sum_{f_{2}=1}^{F}p\left(f_{1},f_{2}\right)\log\left(p\left(f_{1},f_{2}\right)\right),$$

where $p\left(f_{1},f_{2}\right)=\;\frac{{\left|\partial \left(f_{1},f_{2}\right)\right|}^{2}}{\sum_{f_{1}=1}^{F}\sum_{f_{2}=1}^{F}{\left|\partial \left(f_{1},f_{2}\right)\right|}^{2}}$ and $p\left(f_{1},f_{2}\right)$ represent the probability of $\partial \left(f_{1},f_{2}\right)$ in the bispectrum with $f_{1},\;f_{2}=1,\;2,\;\ldots ,F$.

### 4.3. Feature Combination

The EEG frame is decomposed into five subbands’ signals using DWT. The three spike-related features (*A_e_*, *V_sc_*, and *F_i_*) and four entropy features (*pE*, *apE*, *RenE*, and *phE*) are computed from each subband as well as from the full signal. All the features extracted from the six signals are combined to produce the feature vector Z of dimension (*L* + 2) × 7 for each epoch of the EEG signal. Thus, the feature vector derived is used in feature selection, leading to seizure detection with the neural network.

### 4.4. Discriminative Feature Selection

The feature-selection scheme is defined as selecting a subset of significant features for a problem by ranking them according to their importance in the classification model. A GED-based feature selection is implemented to select the dominant features according to the measure of graph centrality [45]. Consider that a set of features Z = {z^(1)^, z^(2)^…, z^(M)^}. An undirected graph G = (H, W) is built, where H is the set of vertices corresponding to the features and W contains the weighted edges connecting the vertices. Consider that the adjacency matrix U for the graph G defines the nature of the weighted edges; each element w_ij_ of W, 1 ≤ ij ≤ M, signifies a pairwise weight [46].

The weight of the graph represents significant criteria associated with class separation for addressing the classification problem. In other words, features are ranked according to their importance classification. On the basis of best practice in feature selection, a combination of two different measures to capture both redundancy and relevance is implemented to define a kernel-based adjacency matrix. The probability distribution is estimated for each feature *z*^(^*^i^*^)^. Consider the Fisher criterion,

(20) $$D_{i}=\frac{{\left|\mu_{i,1}-\mu_{i,2}\right|}^{2}}{\sigma_{i,1}^{2}+\sigma_{i,2}^{2}}\;,$$

where *µ_i,c_* and *σ_i,c_* represent mean and standard deviation, respectively, of the *i*-th feature and samples from the *c*-th class. A larger value of *D_i_* indicates that the *i*-th feature is more discriminative.

For the given class labels, only the features that are related to or lead to these classes are kept. The mutual information is used to rank the features that score high among the features that are highly predictive for the respective class.

(21) $$R_{i}=\sum_{t\in T}\sum_{m\in z^{\left(i\right)}}p\left(m,t\right)\log\left(\frac{p\left(m,t\right)}{p\left(m\right)p\left(t\right)}\right),$$

where *T* is the set of target class labels and *p*(·,·) is the joint probability distribution. Then, the kernel is defined as [47]

(22) $$\Theta =DR^{Tr},\;$$

where (.)*^Tr^* represents the transpose operation, *D* and *R* are *M* × 1 column vectors normalized in [0, 1], and *Θ* results in an *M* × *M* matrix. Another feature-evaluation metric is introduced based on standard deviation [48]. It is a measure of the dispersion of features from their mean and is defined as

(23) $$\rho_{i,j}=\max\left(\sigma_{i},\sigma_{j}\right)\;,$$

where $\sigma_{i}$ represents the standard deviation of *i*th feature and *ρ* is an *M* × *M* matrix with each element *ρ* ∈ [0, 1]. Finally, the adjacency matrix *U* is given by

(24) U=γΘ+(1−γ)ρ, 

where *γ* is a loading coefficient ∈[0, 1]. Each entry *u_ij_* accounts for the degree to which the features *i* and *j* are discriminative when they are considered jointly. At the same time, *u_ij_* is regarded as the weight of the edge connecting the nodes *i* and *j* in graph G. Then, a set of eigenvalues *Λ* and a set of eigenvectors *V* of *U* are computed. The absolute value of principal eigenvector *v*_0_ associated with $\lambda_{0}=\underset{\lambda \in \Lambda }{\max}\left|\lambda \right|$ is used as the weight for ranking the features. The required number of features is selected based on the rank of the individual features.

**Figure 5:**
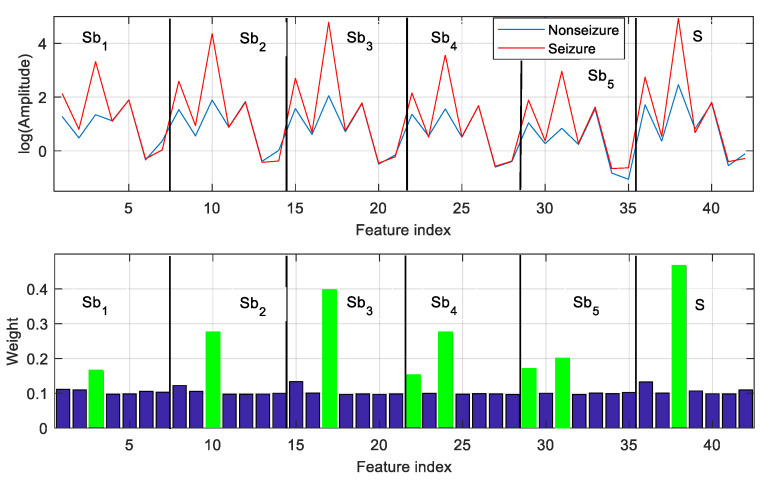
Features and corresponding weights assigned for feature selection. Top panel: feature vectors of length 42 obtained from nonseizure (set A) and seizure (set E) EEG frames. Bottom panel: weight vector derived by the GED approach for feature selection. Each feature is assigned a weight.

*Case study*: Seven features denoted by *Ae*, *Vsc*, *Fi*, *pE*, *apE*, *RenE*, and *phE* are extracted from each of the five subbands as well as the fullband EEG signal. A set of 42 features is obtained from each EEG frame. The features of seizure and nonseizure frames (shown in Figure 3) are presented in the top panel of Figure 5. For better illustration, the logarithmic scale is used to represent feature value. It is observed that the feature vectors obtained from seizure and nonseizure EEG are distinguishable from each other.

The GED-based feature-selection approach assigns a weight to each feature, and the features are ranked based on their weight. Considering the classification task of nonseizure (set A) and seizure (set E), feature selection with GED is applied to the feature vectors extracted from all labeled EEG frames of A and E. The weight vector derived by GED is used to rank the features. Then, the top-ranked features are used for classification. The weight vector used to rank the features in the case of A–E classification is illustrated in the bottom panel of Figure 5. For instance, the weights of the top-ranked 8 features out of 42 are marked in green (bottom panel of Figure 5). It is observed that the feature with higher weight has significant interclass distances (represented by amplitude). The other features make a less significant contribution to A–E classification.

### 4.5. Classification

Artificial neural networks (ANNs) have been widely used in pattern recognition problems for the last few decades [49]. In this study, FfNN is used for the classification of epileptic and nonepileptic EEG signals. Such models are called feedforward because the information only travels forward in the neural network, through the input neuron, then through hidden layers (single or multiple), and finally through the output neuron. It is represented by a combination of many simpler neurons and the connections among them. It works well for nonlinearly separable data. The neuron is the building block of FfNN. When multiple neurons are connected in an effective way, it establishes the required relationship between the neurons to deal with nonlinear data.

A set of selected features is fed into the neural network to perform the classification. The efficient configuration of FfNN to address this problem includes one input, one hidden, and one output layer. The output layer contains one neuron to classify two classes of data. The number of neurons in the hidden later is chosen on the basis of maximizing the performance. On the basis of the selection of discriminative features using the proposed GED-based method, the number of input neurons varies according to the selected number of features. The target values are set to 1 and 0 to represent epileptic and nonepileptic EEG signals, respectively. The hyperbolic tangent sigmoid (HTS) function is used for the input- and hidden-layer transfer function. The Softmax function is assigned to the output layer. The definition of HTS and the Softmax function are given by Equations (25) and (26), respectively.

(25) $$f\left(h_{i}\right)=\;\frac{2}{1+\;e^{-2h_{i}}}-1,\;$$

(26) $$f\left(h_{i}\right)=\;\frac{e^{h_{i}}}{\sum_{j=1}^{N}e^{h_{j}}},$$

where $h_{i}$ represents the hypothesis of the $i^{th}$ neuron and N is the total number of neurons in the output layer. Scaled conjugate gradient backpropagation is used as a network training function to update the weight and bias values of FfNN. Classification accuracy is also assessed using an SVM [50,51] as well as linear discriminant analysis (LDA) [52].

## 5. Experimental Results

As described in Section 3, a well-known EEG dataset is used to conduct the experiments in order to evaluate the performance of the proposed method. The dataset is arranged to evaluate nine different cases of binary class, as shown in Table 2. The feature-selection task, as well as the performance evaluation, is done for each case individually. The details of each set (A–E) are presented in the data description (Section 3).

The data of each set of length 23.6 s are segmented into fixed length frames with reasonable overlapping. The size of the frame plays an important role in seizure detection. A smaller frame size is practically more applicable, whereas it may miss the desired seizure pattern. On the other hand, a larger size of frame could include both seizure and nonseizure events, which can lead to misclassification.

The frame size is set to 10 s with 50% overlapping on the basis of the clinical diagnosis aspect. Each of the five sets having 100 segments of EEG produces 400 frames of 10 s length. Each frame is decomposed by DWT into five mutually orthogonal subbands. The original frame is also kept as an additional subsignal. Hence, each frame contains an array of six subsignals. The features are extracted from each of the six subsignals representing a frame. Three features (*A_e_*, *V_sc_*, and *F_i_*) with which to characterize the spike events and four entropy features (permutation, approximate, Renyi’s, and phase entropy) are extracted from each subsignal. The thus obtained six sets of features are combined to produce the feature vector for a frame. The dominant features are selected using the GED-based supervised method.

Epileptic seizure detection for each frame is a binary classification problem. The FfNN is used to classify any frame as epileptic or nonepileptic. The performance is quantified by classification accuracy (ACC), sensitivity (SEN), and specificity (SPE) measured in percentage (%) defined as follows: ACC = 100 × (*T_P_* + *T_N_*)/(*T_P_* + *T_N_* + *F_P_* + *F_N_*), SEN = 100 × *T_P_*/(*T_P_* + *F_N_*), and SPE = 100 × *T_N_*/(*T_N_* + *F_P_*), respectively, where *T_P_*, *T_N_*, *F_P_*, and *F_N_* represent the number of correctly detected positive, correctly detected negative, erroneously detected positive, and erroneously detected as negative patterns, respectively. In addition to FfNN, the classification performances of LDA and SVM classifiers are also investigated. A *K*-fold (*K* = 5) cross-validation is implemented to accomplish stable and reliable performance of the proposed method [31]. The overall accuracy is the mean of the results obtained from *K* folds. In all cases, 80% of the total frames are used in training, and the remaining 20% are used for validation. For example, in Case 1, there are 800 frames (A–E), among which 640 frames are used in training and 160 EEG frames are used for validation.

The performance is evaluated using the three spike-related features (SrF), four entropy-related features (ErF), with all (seven) combined features (CF), and the combined features with GED (CFGED). Table 3 presents the comparative performance in terms of SEN, SPE, and ACC in percentage (%) averaged over all nine combinations for different feature schemes with three classifiers. With any group of feature combination, the FfNN outperforms SVM and LDA in terms of any of the performance measuring criteria. The higher SEN and SPE of CFGED with FfNN indicate that the proposed method is able to classify both of the classes effectively.

After measuring the performance of different classifiers with various feature groups, the Tukey–Kramer-based post hoc test is performed [53] to test the statistical significance of classification accuracy over different feature groups. According to the results of the post hoc test, FfNN significantly improves performance (in term of classification accuracy) across the feature groups compared with the other classifiers (FfNN versus SVM: *p* < 0.04; FfNN versus LDA: *p* < 0.01). The best performance is accomplished using the CFGED method with FfNN; that is, this study’s proposed method. The other performance matrices (SEN, SPE) also demonstrate the superiority of CFGED with FfNN over other classifiers.

**Figure 6:**
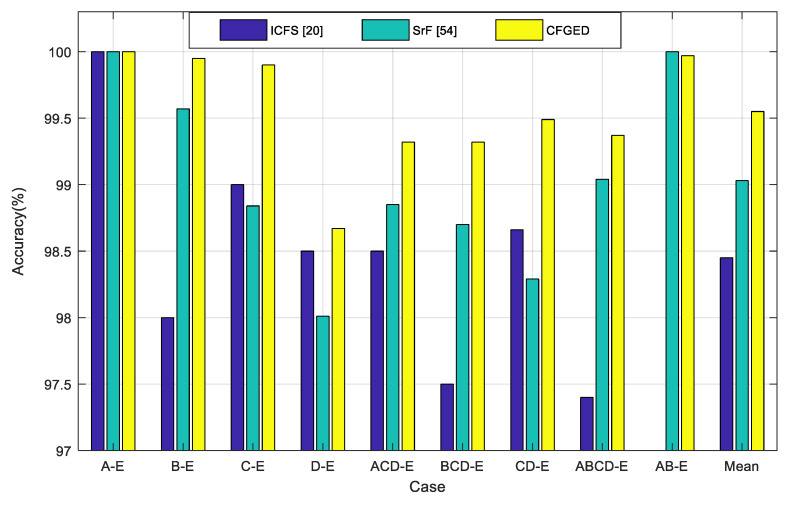
Comparison of the performance of the proposed combined features with graph eigen decomposition (CFGED) method with improved correlation-based feature selection (ICFS) [20] and previous work on spike-related features (SrF) [54].

In the previous work [54], only the three spike-related features (SrF) were used. The EEG data were segmented into epochs of 10 s length and 70% overlapping, whereas 50% overlapping is used in this study. Collectively, the combination of spike-related features with entropy-related features and the GED-based discriminative feature-selection approach improve the classification accuracy of the proposed method. Moreover, the proposed method outperforms the recently developed ICFS [20] algorithm. The comparative results are illustrated in Figure 6. It is evident that the proposed CFGED performs better than the recently developed ICFS method [20] and our previous work with SrF [54]. The average performance (in terms of classification accuracy) across all nine cases confirms the superiority of the CFGED method.

The statistical Friedman test is performed to examine the significance levels of different methods. It is a nonparametric test [55,56] designed to detect differences in methods, including the proposed CFGED. According to the results of the Friedman test, there is a significant main effect of the methods on accuracy (*p* < 0.02). To test the statistical significance of the methods, the Tukey–Kramer-based post hoc test is performed [53]. According to the results of the post hoc test, the CFGED method significantly improves the performance of classification across the nine cases compared with the other methods (CFGED versus SrF [54]: *p* < 0.02; CFGED versus ICFS [20]: *p* < 0.007). It is observed that the maximum performance is achieved using the CFGED method.

**Figure 7:**
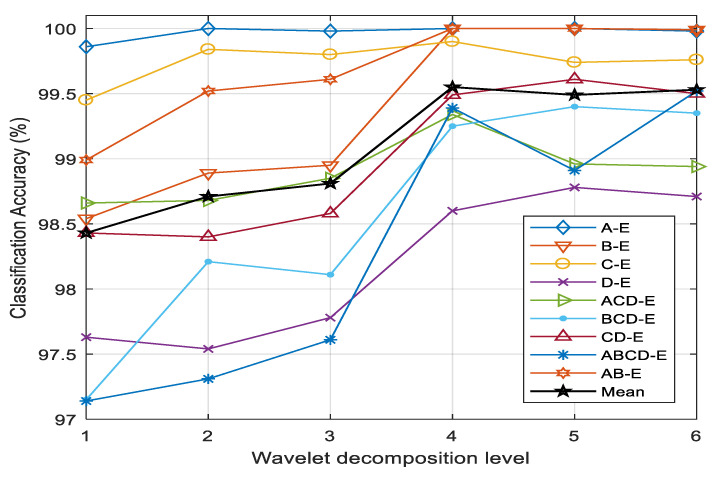
Classification accuracies of different cases and the mean accuracy as a function of wavelet decomposition levels to generate the subbands.

The number of subbands obtained by applying DWT to EEG is a significant factor of classification performance. It depends in turn on the level of wavelet decomposition. The classification accuracies of nine cases with different levels of wavelet decomposition are illustrated in Figure 7. It is evident that the highest average classification accuracy is accomplished with four levels. In most of the cases, the accuracy increases up to the fourth level and remains almost constant at levels greater than four. The subbands obtained by a wavelet decomposition level higher than four have a negligible impact on the performance of EEG classification into seizure and nonseizure signals. The higher-order subbands contain lower-frequency components, which do not carry sufficient variance to detect seizure events in EEG signals. Considering the performance issue, four-level wavelet decomposition (five subbands) is implemented in this study.

## 6. Discussion

In this study, the features of two different domains are combined, and an effective feature-selection approach is implemented to detect seizure events in EEG signals. The first category has three features that represent the fluctuation characteristics, and the second category includes four different entropies that illustrate the complexity of the signal. The combined feature space has seven features corresponding to each EEG frame. In total, seven different classification problems are considered. The performance with the selected features is evaluated using three supervised classifiers, namely, SVM, LDA, and FfNN, with a fivefold cross-validation technique. The simulation results show that the proposed method has effective performance in epileptic seizure classification.

A number of previous works are summarized in this section with results (in terms of classification accuracy) to realize their performance in seizure detection. The results of several existing works in which the Bonn university dataset is used for performance evaluation are compared with the proposed CFGED method, as presented in Table 4. Among the algorithms, the mean accuracy of SrF [54] (averaged over nine cases) outperforms all the previously developed algorithms, whereas the average classification accuracy of the proposed method is higher than that of SrF [54]. It is necessary to consider the average accuracy over the possible cases to compare the overall performance of any method. Although the average classification accuracy of [14,20] are similar (98.45%), the evaluation case CD–E is included in [20], but not in [14]. Sets C and D are recorded from epileptic subjects while seizure is not present. On the other hand, set E is recorded from epileptic subjects at the time of seizure. It is often difficult to distinguish set C and set D from seizure EEG signals taken from set E. The issue is evaluated by case 7 (CD–E) with a classification accuracy of 99.49% using the CFGED method. The same evaluation case (CD–E) is considered in [18] and achieves an accuracy of 98.80%.

The proposed method achieves the highest average accuracy over nine different evaluation cases. Notably, the maximum number binary classification cases are considered in this study. The highest classification accuracy of 100% is achieved with CFGED in case 1 (A–E), case 2 (B–E), and case 9 (AB–E). The lowest accuracy is 98.60% in case 4 (D–E), which is still 0.10% greater than that of ICFS [20] (98.50%). The accuracies for case 4 (D–E) of all other methods mentioned in Table 4 are lower than the accuracy achieved by ICFS [20].

Only one case, A–E, is considered in [12,16,17], with classification accuracies of 99.44%, 99.56%, and 99.20%, respectively, whereas the accuracy of the CFGED method is 100% for case A–E. The accuracy of case 1 (A–E) and case 9 (AB–E) achieved by the method SrF [54] is 100%, whereas the average performance of the proposed method is higher than that of SrF [54]. Nine different evaluation cases are also considered in [6], which attains 97.15% verage accuracy, which is 2.40% less than the classification accuracy achieved by CFGED. Considering the overall situation, the average classification accuracy of the proposed CFGED method outperforms all the recently developed algorithms with minimum standard deviation. In CFGED, the EEG frame is decomposed into narrowband signals to localize the feature in frequency scale. The subband features used in CFGED are very effective for epilepsy detection, yielding higher accuracy. Two categories of features are combined in the proposed method. The spike-related features capture the transient events in EEG signals that are caused mostly by seizure events. On the other hand, the entropy-based features measure the seizure-related complexity of the signals. Three spike-based features and four entropy features are collectively used in this study to detect seizure events, and hence improvement in the classification performance is achieved.

The number of selected features is another factor that affects the performance of the proposed method. The features are ranked based on the performance of any feature in the discrimination between the classes. Each feature is assigned a weight using a GED-based feature-selection method. The features with higher weights are more discriminative and have greater potential for seizure/nonseizure event classification. The inclusion of low-rank features reduces the class discrimination that leads to a decrease in the classification accuracy. A number of high-rank features are selected for seizure detection in an effort to maximize the performance.

**Figure 8:**
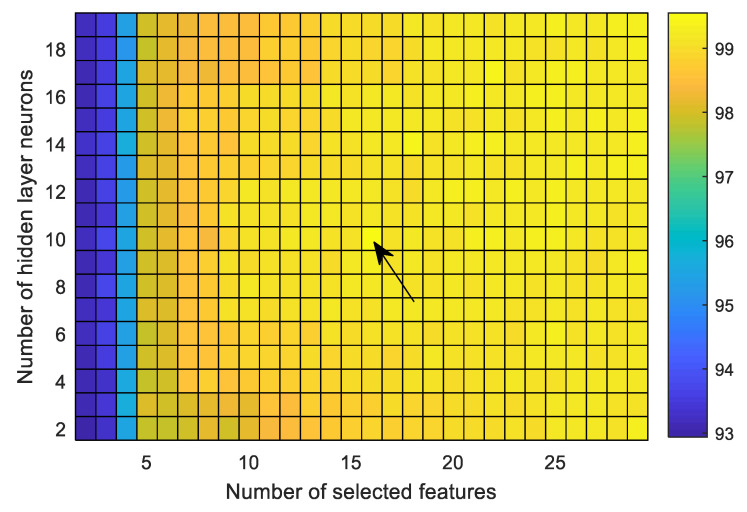
Average performance over nine cases as a function of the number of selected features (using GED) and number of neurons used in the hidden layer of FfNN. The maximum average accuracy is achieved with 18 selected features and 10 hidden neurons.

Another important parameter that influences the performance is the number of neurons used in hidden layer of FfNN. This issue has yet to be investigated properly. It depends mostly on the different factors, including feature dimension as well as its characteristics, the target outputs, and the learning algorithm. It has already been mentioned that the average accuracy rather than that of the individual case is more effective for comparing the performance. To obtain the maximum average accuracy, a grid-search method is used to select the number of features as well as the hidden neurons. Figure 8 illustrates the average classification accuracies over the nine cases as a function of the number of selected features as well as the number of hidden neurons. The number of features and hidden neurons is minimized to obtain maximum average accuracy, aiming to reduce the computation complexity. It is observed that the maximum average accuracy is obtained with 16 selected features and 10 hidden neurons. In this study, all the experiments are conducted using these selected parameters to perform the comparison with other methods.

Besides different entropies, the features to characterize the spike events in EEG are also used in this study. A potential feature-selection approach is implemented to enhance the classification performance of the seizure/nonseizure event using scalp EEG. The features are extracted from the EEG signal as well as its different subbands obtained by DWT. The raw feature vector has high dimensionality. Various features might be inaccurate and mislead the classifier, leading to a weakening of the overall system performance [57].

**Figure 9:**
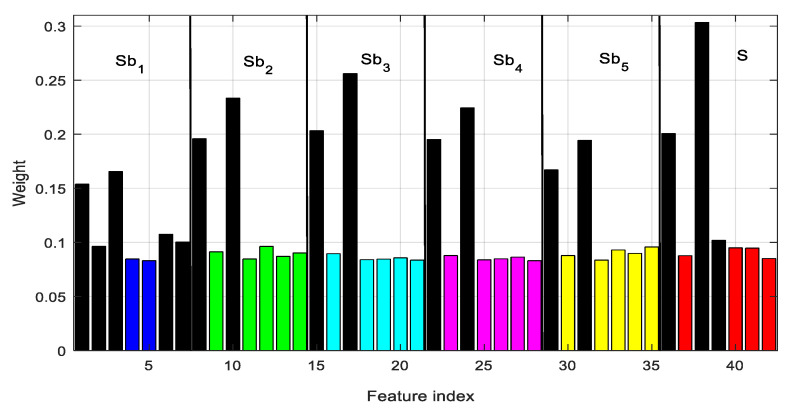
The weight of individual features extracted from different subbands (indicated by different colors). The 16 features are selected (black color) from different subbands according to their weights. Sb_1_…Sb_5_ represent five subbands and fullband EEG signal (before subband decomposition) for case D–E.

A discriminative subset of features extracted from EEG is more suitable to correctly classifying the seizure event, whereas the others must be removed to reduce performance degradation. The selection of the subset of appropriate features also requires the removal of the irrelevant or redundant elements in the feature vector. It also reduces overfitting of training time in machine learning. A GED-based supervised feature-selection method is implemented here. The weights assigned by GED to each of 42 features of an EEG epoch of case 4 (D–E) are shown in Figure 9. It is observed that the 38th feature has the maximum weight, and hence is the top-ranked feature. The feature vector is sorted based on the weight assigned to each one. The top-ranked 16 features are selected (from the sorted feature vector) to be used in seizure detection. Another interesting phenomenon is the effectiveness of using subband features. The 16 features are selected from different subbands rather than from the original EEG. Each subband produces seven features. Five, two, two, two, and two features are selected from subbands Sb_1_, Sb_2_, Sb_3_, Sb_4_, and Sb_5_, respectively, and the remaining three from a fullband EEG signal (S). This provides evidence that, without employing the subband features, the seizure-detection performance is likely to be reduced. The subband features represent discriminative components localized in narrowband signals.

## 7. Conclusions

Automatic detection of epileptic seizure in EEG is a challenging task owing to its subject dependency and lack of available training data. A publicly available EEG dataset is used to evaluate the efficiency and effectiveness of the proposed method. The main contribution in this study includes the development of an effective feature vector to detect the epileptic seizure event in EEG within a reasonable time duration and a discriminative feature-selection scheme. The EEG is decomposed into subband signals using DWT to compute subband features. Two categories of features are extracted from each of the subbands as well as from the fullband signal: (1) three features that characterize the signal’s spike events and (2) four different entropies that quantify the complexity carried by the signal. The combination of the features obtained from the subbands and the EEG signal itself yield the high-dimensional feature vector. All the features are not equally important to perform seizure/nonseizure classification. A discriminative subset of features is selected from the feature vector using a GED-based approach. The irrelevant features are removed from the feature vector to defend the degradation of classification performance. The selected features are fed to FfNN to recognize the seizure event in EEG. The performances of state-of-the-art algorithms are compared to the proposed CFGED method. The experimental results provide evidence that the proposed approach outperforms other methods of classifying seizure and seizure-free EEG signals. The use of the mentioned features derived on the basis of the underlying characteristics of epileptic EEG as well as the selection of discriminative features make the proposed CFGED method robust, with high classification accuracy. The results obtained are promising and applicable in the implementation of an automatic seizure-detection system. The related research community will benefit greatly from the proposed seizure and nonseizure classification of EEG signals collected from the scalp. Considering performance, it will assist neurologists in detecting epilepsy, greatly reduce examination time, and increase their efficiency. There are many future directions related to the proposed work. Although the proposed seizure-detection method achieves good performance on an experimental benchmark dataset, its clinical validation and examination of its suitability for deployment in a clinical setting are considered future work.

## Figures and Tables

**Table 1:** Summary of the electroencephalography (EEG) dataset used in this study.

Subjects	Healthy Subjects		Epileptic Subjects		
	Set A	Set B	Set C	Set D	Set E
Alternative representation	Set Z	Set O	Set N	Set F	Set S
Patients state	Eyes open and awake (normal)	Eyes closed and awake (normal)	Seizure-free (interictal)	Seizure-free (interictal)	Seizure activity (ictal)
Number of epochs	100	100	100	100	100
Time duration (s)	23.6	23.6	23.6	23.6	23.6
Electrode type	Surface	Surface	Intracranial	Intracranial	Intracranial
Electrode placement	International 10–20 System	International 10–20 System	Opposite to epileptogenic zone	Within epileptogenic zone	Within epileptogenic zone

**Table 2:** Different cases derived from the dataset for epileptic seizure classification.

Class	Case								
	1	2	3	4	5	6	7	8	9
Class 1 (nonseizure)	A	B	C	D	ACD	BCD	CD	ABCD	AB
Class 2 (seizure)	E	E	E	E	E	E	E	E	E

**Table 3:** Comparative performance in terms of average sensitivity (SEN), specificity (SPE), and classification accuracy (ACC) measured in percentage (%) over all nine cases with different groups of features and classifiers. SrF, spike-related features; ErF, entropy related features; CF, combined features; CFGED, combined features with graph eigen decomposition; FfNN, feedforward neural network; SVM, support vector machine; LDA, linear discriminant analysis.

Classifier	SrF			ErF			CF			CFGED		
	SEN	SPE	ACC	SEN	SPE	ACC	SEN	SPE	ACC	SEN	SPE	ACC
FfNN	99.45	98.86	99.03	99.05	98.25	98.85	99.48	98.75	99.32	100.00	99.46	99.55
SVM	98.81	98.10	98.47	98.60	98.08	98.24	99.42	99.17	99.29	99.77	99.21	99.39
LDA	98.35	97.86	98.15	98.20	96.95	97.91	99.08	98.12	98.69	98.95	98.10	98.72

**Table 4:** Comparison of performance (in terms of classification accuracy) of the proposed method with recently developed algorithms for seizure classification. LS, least square; GP, genetic programming; KNN, *k*-nearest numbers; ICFS, improved correlation-based feature selection; SODP, second-order difference plot; EMD, empirical mode decomposition.

Reference	Methods	Cases	Accuracy (%)
Ubeyli et al. [16], 2010	LS-SVM model	A–E	99.56
Wang et al. [12], 2011	Wavelet packet entropy	A–E	99.44
Guo et al. [17], 2011	GP with KNN classifier	A–E	99.2
Y. Kumar [14], 2014	Fuzzy approximate entropy and SVM	A–E	100
		B–E	100
		C–E	99.6
		D–E	95.85
		ACD–E	98.15
		BCD–E	98.22
		ABCD–E	97.38
		Mean ± STD	98.45 ± 1.53
N. S. Tawfik [15], 2015	Weighted permutation entropy (WPE) and SVM	A–E	98.5
		B–E	85
		C–E	93.5
		D–E	96.5
		Mean ± STD	93.37 ± 5.95
T. S. Kumar [19], 2015	Gabor filter with KNN	CD–E	98.3
R. B. Pachori [7], 2014	SODP with EMD	CD–E	97.75
M. Mursalin [20], 2017	ICFS with random forest classifier	A–E	100
		B–E	98
		C–E	99
		D–E	98.5
		ACD–E	98.5
		BCD–E	97.5
		CD–E	98.66
		ABCD–E	97.4
		Mean ± STD	98.45 ± 0.84
I. Ullah [18], 2018	Convolution neural network (CNN)-based deep learning	A–E	99.9
		B–E	99
		C–E	98.8
		D–E	98.1
		AB–E	97.4
		CD–E	98.8
		Mean ± STD	98.67 ± 0.85
S. Raghu [6], 2019	Matrix-determinant-based features with multilayer perceptron (MLP)	A–E	99.45
		B–E	96.06
		C–E	97.6
		D–E	97.6
		AB–E	97.1
		AC–E	96.5
		CD–E	96.85
		ACD–E	96
		ABCD–E	97.2
		Mean ± STD	97.15 ± 1.04
Hassan et al. [54], 2019	Multiband implementation of three spike-related features (SrF)—*A_e_*, *V_c_*, and *F_i_* with FfNN	A–E	100
		B–E	99.57
		C–E	98.84
		D–E	98.01
		ACD–E	98.85
		BCD–E	98.7
		CD–E	98.29
		ABCD–E	99.04
		AB–E	100
		Mean ± STD	99.03 ± 0.70
Proposed method (CEGED)	Multiband implementation of three spike-related features (SrF) and four entropy features with GED-based feature selection with FfNN classifier	A–E	100
		B–E	100
		C–E	99.9
		D–E	98.6
		ACD–E	99.34
		BCD–E	99.25
		CD–E	99.49
		ABCD–E	99.39
		AB–E	100
		Mean ± STD	99.55 ± 0.47
